# Treating Iron Deficiency (ID) Anemia in Heart Failure (HF) Patients with IV Iron: A Meta-Analysis

**DOI:** 10.7759/cureus.41895

**Published:** 2023-07-14

**Authors:** Fredrick M Ogugua, Francisco A Aguilar, Abdulrahman Gamam, Muhammad Haisum Maqsood, Tae Kyung Yoo, Fedi Kasmi, Oubada AlKowatli, Kevin Lo

**Affiliations:** 1 Cardiology, University of Illinois Chicago, Chicago, USA; 2 Internal Medicine, Einstein Medical Center Philadelphia, Philadelphia, USA; 3 Internal Medicine, Minneapolis Heart Institute, Minneapolis, USA; 4 Internal Medicine, Lincoln Medical Center, New York, USA; 5 Medicine, MetroWest Medical Center, Framingham, USA; 6 Internal Medicine, Sheikh Khalifa Hospital, Ajman, ARE; 7 Internal Medicine, Advocate Illinois Masonic Medical Center, Chicago, USA

**Keywords:** ischemic cardiomyopathy, intravenous iron supplement, hematic values, heart failure hospitalization, erythropoietin

## Abstract

Findings on the effects of iron on heart failure (HF) hospitalizations and mortality among patients with iron deficiency (ID) and HF remain conflicting across different studies. We performed a meta-analysis of clinical trials assessing the clinical, hematic and cardiovascular benefits of treating ID in HF patients.

We completed a systematic search for studies comparing IV iron to placebo in HF patients with ID. The primary outcomes were rates of HF hospitalization and all-cause mortality. Secondary outcomes included change in hematic values, New York Heart Association (NYHA) class and ejection fraction. We applied a random-effects model with planned sensitivity analyses of studies with skewed effect sizes.

Nine studies were included with a total of 2,261 patients. Analysis revealed that treatment of HF patients with IV iron replacement significantly reduced the odds of HF hospitalization (odds ratio (OR): 0.44; 95% confidence interval (CI): 0.24 to 0.78; p=0.005, I^2^=67%),) but did not significantly impact all-cause mortality compared to placebo (OR: 0.89; 95%, CI: 0.67 to 1.19; p=0.44, I^2^: 0%). Analysis showed that IV iron treatment group had significantly higher serum ferritin, transferrin saturation and hemoglobin (Hb) levels. They also had lower NYHA class -1.90 (95% CI (-2.91 to -0.89); p<0.001, I^2^:89%) with higher ejection fraction 0.50 (95% CI (0.09 to 0.90) p=0.016, I^2^:86%).

Treatment with IV iron in HF patients with ID is associated with a significant reduction of HF hospitalization but no effects on all-cause mortality. There were also significant increases in hematic values and ejection fraction with a reduction in NYHA class.

## Introduction and background

Heart failure (HF) and its associated cost continues to be a huge burden on the United States (US) healthcare system, with cardiovascular morbidity, recurrent hospitalizations and mortality being major contributors [1]. In the US, 1 in 50 adults have HF and, in 2014, about 1 million hospitalizations were due to HF, with a total estimated expenditure of $11 billion [1,2].

Iron deficiency (ID) is common in HF patients with a reported prevalence of about 40-70% [3-5]. The underlying mechanism of ID in HF is incompletely understood, but factors like inflammation, reduced intake, impaired absorption, downregulation of iron transporters, and chronic gastrointestinal blood loss from use of antiplatelet agents or anticoagulants have been proposed to explain the relationship [6].

It has been proven that ID is associated with decreased exercise capacity, low quality of life and is independently associated with poor prognosis and mortality in HF patients [7-9]. Due to these findings, it becomes important to address ID in HF patients. Multiple studies have shown that the treatment of ID using IV iron replacement improves hematic values, functional capacity, and quality of life in HF patients [10,11]. These findings led the European Society of Cardiology (ESC) and American College of Cardiology/American Heart Association (ACC/AHA), to publish a Class II A, Level of Evidence A-B recommendation to treat ID in HF patients [12,13]. Despite these recommendations, there has been suboptimal uptake in practice, likely due to a variety of factors but partially driven by conflicting results from various studies.

van Veldhuisen et al. and Yeo et al. in their studies showed that IV iron therapy did not reduce rates of HF hospitalization or impact mortality, whereas, studies by Comín-Colet et al. and Ponikowski et al. demonstrated that the use of IV iron reduces HF hospitalization. [14-17]

Due to the lack of uptake in clinical practice and mixed results, we performed a meta-analysis of clinical trials assessing the rates of HF hospitalization, cardiovascular, and mortality benefit of treating ID using IV iron in HF patients. 

## Review

Methods

Study Design and Inclusion Criteria

Electronic searches:We performed a comprehensive search of MEDLINE, CINAHL, and Cochrane CENTRAL for clinical trials until September 2021 [18]. We identified clinical studies that reported rates of clinical outcomes of interest among HF patients with ID treated with IV iron with or without erythropoietin (EPO) versus placebo.

Types of studies and participants: We included randomized (RCT) and non-randomized clinical trials (nRCT) comparing therapy with IV iron with or without (EPO) versus placebo in HF patients with ID regardless of left ventricular ejection fraction (LVEF), aged 18 years and above. ID was defined as ferritin < 100 ng/mL, or ferritin between 100 and 300 ng/mL with a transferrin saturation (TSAT) < 20%. We included studies reported as full‐text and excluded those published as abstracts only. We also excluded observational studies, case reports, case series, review articles, letters to the editor, comments, and editorials.

Types of interventions: Different types of IV iron were used in the studies as intervention (with or without EPO), namely; ferric carboxymaltose, iron sucrose, iron isomaltoside, iron dextran and erythropoietin.

Primary and secondary outcome: Co-primary outcomes were HF hospitalization and all-cause mortality rates. Secondary outcomes were changes in hematic values and cardiovascular parameters namely; New York Heart Association (NYHA) class and LVEF.

Data Collection and Analysis 

Five authors independently reviewed the title and abstract of the studies identified in the primary search and excluded studies that did not address the research question, based on pre-specified inclusion and exclusion criteria. The full text and bibliographic section of the remaining articles were reviewed for relevant content. Any discrepancy in article selection was resolved by consensus and in discussion with co-authors. Three authors (F.O., A.G., O.K.) independently extracted details regarding study and patient characteristics, treatment strategy, duration of follow-up and the total number of clinical endpoints of interest. Data of interest were entered into an Excel spreadsheet and subsequently verified by two other authors (M.H., F.A.). Data was entered into Revman version 5.3 for meta-analysis using the random effects model. Odds ratio (OR) with 95% confidence intervals (CIs) for all outcomes were used to present the pooled effect size of the individual studies for the primary outcomes. Using a random effects model on MedCalc version 20, we analyzed the secondary continuous outcomes of the hematic parameters, NYHA class and LVEF inputting mean and standard deviations yielding the SMD and 95% CI. The results of the pooled effect size were considered to be statistically significant at p < 0.05.

Assessment of risk of bias in included studies: Using the criteria outlined in the Cochrane Handbook for Systematic Reviews of Interventions and by Wood et al., we assessed the risk of bias of selected RCTs [18,19]. Conversely, the Newcastle-Ottawa scale was used to assess the quality of the chosen nRCT [20]. Four investigators (F.O., A.G., M.H., F.A.) carried out the quality assessment independently, and a fifth (K.L.) investigator helped resolve disagreements.

Assessment of heterogeneity and reporting bias: The impact of heterogeneity on our meta-analysis was quantified using the I^2^ statistic. An I^2^ statistic of >50% was indicative of significant heterogeneity. A random effects model was used throughout to suppress bias introduced by significant heterogeneity. Reporting bias of included studies was also examined using Cochrane Revman version 5.3.

Sensitivity analyses: Sensitivity analyses were carried out by running separate analyses excluding studies with skewed effect sizes compared to the rest and watching the effect on the heterogeneity. Studies with fundamental differences in methodology were analyzed both together and separately as part of these sensitivity analyses. 

Results

Results of the Search 

**Figure 1:**
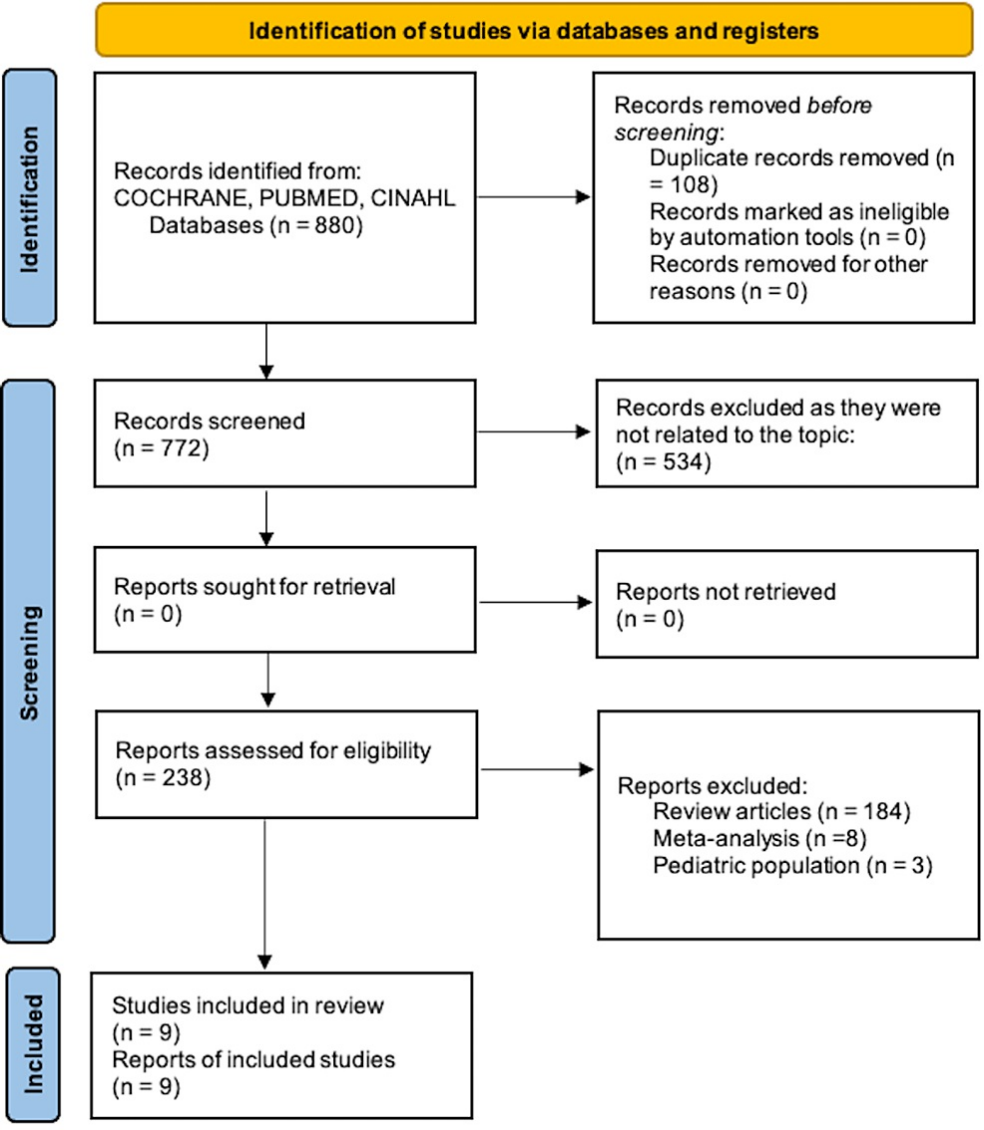
Illustration of study selection flow diagram

The initial search yielded 880 citations. 108 were excluded because they were duplicate publications. After the initial screening of 772 titles and abstracts based on the aforementioned criteria, 43 citations underwent secondary review, and among them, nine published studies met the eligibility criteria for inclusion [10,11,14-17,21-23]. The study selection process is illustrated in Figure 1.

Included Studies 

A total of 2,261 patients were included in the nine selected studies, and the characteristics of the studies are presented in Table 1. There was no inter-rater disagreement for the extracted data. Eight studies were RCTs, while one study was nRCT. Most studies were multicenter, mostly located in the United States, Europe and Australia, except those by Silverberg et al., Comín-Colet et al., and Toblli et al., which were single-center studies [10,16,21]. Sample sizes across studies varied from small to large sample sizes. The mean age of patients was 68.7 years. There were no significant differences in the baseline characteristics of the groups. Follow-up time ranged from 12 to 52 weeks. A total of 1,210 patients were in the intervention group while 1,051 were in the placebo group. The inclusion criteria for the studies are listed in Table 1. Study quality overall was strong based on adherence to the reporting principles of the CONSORT statement [24]. All patients in the included trials had established chronic HF and NYHA Class II-III except Yeo et al., which did not report NYHA class. [15]

 

**Table 1:** Baseline characteristics of included studies LVEF: Left ventricular ejection fraction; NYHA: New York Heart Association; Hb: Hemoglobin; HF: Heart failure; TSAT: Transferrin saturation; CrCl: Creatinine clearance; CRP: C-reactive protein; 6MWT: 6-minute walk test; MLHFQ: Minnesota living with heart failure questionnaire; pVO_2_: Normal mixed venous oxygen tension; PGA: Patient global assessment; KCCQ:  Kansas City cardiomyopathy questionnaire; EQ-5D: European quality of life 5D; CKD: Chronic kidney disease; QoL: Quality of life questionnaire; VAS: Visual analogue scale; CV: Cardiovascular death

Study name	Patients (IV iron/Control)	Mean age (IV iron/Control)	Iron product and dosing	Follow up period	Inclusion criteria	Endpoints	LVEF	NYHA class
Toblli et al. [10]	20/20	76/74	Iron sucrose, 200 mg/ wk	24 weeks	Hb 12.5 g/dL (men) or Hb 11.5 g/dL (women), ferritin < 100 ng/mL, TSAT 20%, CrCl 90 mL/min	NT-proBNP, CRP, 6MWT, MLHFQ, LVEF, HF hospitalization	<35%	II-IV
Okonko et al. [11]	24/11	64/62	Iron sucrose, 200 mg/ wk	18 weeks	pVO2/kg 18 mL/kg/min, Hb <12.5 or 12.5-14.5 g/dL, ferritin <100 g/L or 100-300 g/L with TSAT <20%	NYHA class, LVEF, pVO_2_, PGA, MLHFQ, CRP, adverse events	<45%	II-III
van Veldhuisen et al. [14]	86/86	63/64	Ferric carboxymaltose 500 mg or 1 g weekly	24 weeks	NT-proBNP >400 pg/mL, Hb <15 g/dL, ferritin <100 ng/mL or 100-300 ng/mL with TSAT<20%	pVO_2_, NYHA class, PGA, NT-proBNP, adverse events	45%	II-III
Yeo et al. [15]	25/25	61.1/64	Ferric carboxymaltose 1000 mg	12 weeks	21 yrs and above, Hospitalized for acute HF, serum ferritin <300 ng/mL if TSAT is <20%) and Hb ≤14 g/dL, Able to complete the 6-MWT	6-MWT, QoL, visual analogue scale, NYHA class	Any	Miscoded- not included
Comín-Colet et al. [16]	27/38	74/74	Iron sucrose 200 mg/ week for 5-6weeks + erythropoietin	66 weeks	Hb <13.0 g/dL in men; Hb <12.0 g/dL in women, CKD II-III or creatinine <3mg/dl	NT-proBNP, Hb, NYHA class, HF hospitalization, all-cause mortality, composite of HF hospitalization and all-cause mortality	<35%	III-IV
Ponikowski et al. [17]	558/550	71.2/70.9	Ferric carboxymaltose 500 to 1000 mg every 6 weeks based on weight and Hb levels	52 weeks	Hospitalized for acute HF, LVEF<50%	Composite of HF hospitalization and CV death	<50%	I-IV
Silverberg et al. [21]	16/16	75.3/72.2	Iron sucrose 200 mg every 2 weeks + erythropoietin	54 weeks	Hb 10 to 11.5 g/dl	NYHA, serum creatinine, days in hospital, Hb, ferritin LVEF, HF hospitalization, all-cause mortality	<40%	III-IV
Anker et al. [22]	304/155	68/67	Ferric carboxymaltose 200 mg weekly for 4 weeks	26 weeks	Hb 9.5-13.5 g/dL, ferritin <100 g/L or 100-299 g/L with TSAT <20%	NYHA class, 6MWT, PGA, EQ-5D, KCCQ, adverse events	<40-45%	II-III
Ponikowski et al. [23]	150/151	69/70	Ferric carboxymaltose 500 mg or 1 g weekly	52 weeks	NT-proBNP >400 pg/mL, Hb <15 g/dL, ferritin <100 ng/mL or 100-300 ng/mL with TSAT<20%	NYHA class, 6MWT, PGA, KCCQ, EQ-5D, adverse events	45%	II-III

HF Hospitalization

Eight studies out of included nine reported rates of HF hospitalization with 2,229 participants (1,194 IV iron group, 1,035 placebo group) [10,11,14-17,22,23]. The placebo group had 389 hospitalizations compared to 264 in the IV iron group. Overall, compared to placebo, the IV iron group had significantly reduced odds of HF hospitalization in iron-deficient HF patients (OR: 0.44; 95% CI: 0.24 to 0.78; p=0.005) (Table 2). We repeated our analysis by comparing studies that used IV iron only vs placebo. Hence, we excluded the study by Comín-Colet et al. and our results remained statistically significant (OR: 0.54; 95% CI: 0.32 to 0.9; p= 0.02).

**Table 2:** Analysis comparing events for HF hospitalization Heterogeneity Tau^2^ = 0.37; Chi^2^ = 21.18, df = 7 (p=0.004); I^2^ = 67% Test for overall effect: Z = 2.80 (P=0.005) HF: Heart failure; CI: Confidence interval; df: Degree of freedom; OR: Odds ratio

Study	Year	IV Iron Therapy		Placebo		Weight (%)	OR
		Events	Total	Events	Total		M-H, Random, 95% CI
Toblli et al. [10]	2007	0	20	5	20	3.3	0.07 (0.00, 1.34)
Okonko et al. [11]	2008	1	24	2	11	4.3	0.20 (0.02, 2.43)
van Veldhuisen et al. [14]	2017	13	86	13	86	15.8	1.00 (0.43, 2.30)
Yeo et al. [15]	2018	9	24	5	25	11	2.40 (0.60, 8.65)
Comín-Colet et al. [16]	2009	7	27	29	38	12.3	0.11 (0.03, 0.34)
Ponikowski et al. [17]	2020	217	558	294	550	22.6	0.55 (0.44, 0.70)
Anker et al. [22]	2009	7	305	9	154	13.8	0.38 (0.14, 1.04)
Ponikowski et al. [23]	2015	10	150	32	151	16.9	0.27 (0.13, 0.56)
Total		264	1,194	389	1,035	100	0.44 (0.24, 0.78)

All-Cause Mortality

Eight studies out of included nine reported rates of mortality with 2,221 participants (1,190 IV Iron group, 1,031 Placebo group) reported rates of all-cause mortality. [11,14-17,21-23] The placebo group had 117 cases of mortality compared to 104 in the IV Iron group. Overall, the use of IV iron did not significantly impact rates of all-cause mortality compared to placebo in HF patients with ID (OR: 0.89; 95% CI: 0.67 to 1.19; p= 0.44) [Table 3]. The exclusion of studies by Comín-Colet et al. and Silverberg et al. that include the use of EPO did not influence the lack of mortality difference (OR: 0.92; 95% CI: 0.68 to 1.24; p= 0.60).

**Table 3:** Analysis comparing events for all-cause mortality Heterogeneity Tau^2^ = 0.00; Chi^2^ = 5,68, df = 7 (p=0.58); I^2^ = 0% Test for overall effect: Z = 0.88 (P=0.44) CI: Confidence interval; df: Degree of freedom; OR: Odds ratio

Study	Year	IV Iron Therapy		Placebo		Weight (%)	OR
		Events	Total	Events	Total		M-H, Random, 95% CI
Okonko et al. [11]	2008	1	24	0	11	0.8	1.47 (0.06, 38.91)
van Veldhuisen et al. [14]	2017	0	86	4	86	1	0.11 (0.01, 2.00)
Yeo et al. [15]	2018	1	24	0	25	0.8	3.26 (0.13, 83.90)
Comín-Colet et al. [16]	2009	8	27	13	38	7.3	0.81 (0.28, 2.35)
Ponikowski et al. [17]	2020	77	558	78	550	71.8	0.97 (0.69, 1.36)
Silverberg et al. [21]	2001	0	16	4	16	0.9	0.08 (0.00, 1.71)
Anker et al. [22]	2009	5	305	4	154	4.7	0.63 (0.17, 2.36)
Ponikowski et al. [23]	2015	12	150	12	151	12.7	0.85 (0.38, 1.91)
Total		104	1190	117	1031	100	0.89 (0.67, 1.19)

Hematic Values 

Regarding hematic values, the pooled standardized mean difference (SMD) of the serum ferritin levels between treatment and control groups was 1.45 95% CI (0.77 to 2.13) p<0.001 based on random effects model with 97% heterogeneity. Removal of the outlier study by Yeo et al. only reduced the heterogeneity to 96%, but the results remained statistically significant [15]. The SMD for transferrin saturation in % was 0.41; 95% CI (0.04 to 0.78), p=0.028, heterogeneity was 90%. In a sensitivity analysis, removing the outlier Toblli et al. reduced the heterogeneity to 86% and the results remained statistically significant. The SMD for Hb levels was 0.79 95% CI (0.42 to 1.15) p<0.001 with heterogeneity of 92%. Removal of the outlier Toblli et al. reduced heterogeneity to 88% but results remained statistically significant [10]. Overall, treatment with IV iron was associated with a significant increase in serum ferritin, transferrin saturation and hemoglobin (Hb) levels, but all pooled analyses had high heterogeneity.

Cardiovascular Parameters

The SMD for NYHA class was -1.90 (95% CI (-2.91 to -0.89); p<0.001) with heterogeneity of 89%. A sensitivity analysis removing the study by Okonko et al. (due to having the shortest follow-up period of just 4-18 weeks) yielded SMD of -2.60 with (95% CI (-2.91 to -0.89); p<0.001) with 0% heterogeneity [11]. Lastly, the SMD for ejection fraction (EF) was 0.50 with 95% CI (0.09 to 0.90); p=0.016 heterogeneity 86%. After adjusting for the outlier study of Toblli et al., which included patients with lower EF (<35%), SMD was 0.58 with (95% CI (-0.115 to 1.269)), the heterogeneity dropped to 69% but this was no longer statistically significant p=0.135 [10]. Overall, the use of IV iron compared to placebo led to a statistically significant reduction in NYHA class but with a weaker effect on the improvement of EF. All pooled analyses had significant heterogeneity.

Heterogeneity

I^2^ statistic during analysis for hematic values, cardiovascular parameters and rates of HF hospitalization were all higher than 50% likely, indicating significant heterogeneity. However, given the binary nature of our measured outcomes, we anticipate that the impact of the observed high heterogeneity is likely less significant. At any rate, we employed a random effects model in our analysis. Additionally, we examined the heterogeneity by performing sensitivity analyses.

Discussion

Anemia and ID often co-exist in HF patients and iron replacement therapy has been studied with variable results [17,25,26]. The main finding in this meta-analysis of nine studies is that therapy with IV iron in iron-deficient patients with HF is associated with significantly lower rates of HF hospitalization. Similarly, IV iron therapy was significantly associated with improved NYHA class, EF and hematic values, including ferritin level, transferrin saturation, and Hb. However, IV iron therapy did not statistically change rates of all-cause mortality.

Lower Rate of Hospitalization in IV Iron Group

Our study showed decreased hospitalization rates among iron-deficient HF patients who had received therapy with IV iron. Our finding is similar to recent meta-analyses by Zhang et al. and Anker et al. [27,28]. Our study is unique in that, intervention was limited to IV iron +/- EPO and is the largest number of meta-analyzed HF patients [26,27,29].

Multiple studies have identified ID as a comorbid condition independently associated with increased rates of re-hospitalization and mortality in HF patients [7-9]. Our study and more recent research have shown that treatment of ID in HF patients reduces HF hospitalization rates [17]. Other benefits include increased exercise capacity and quality of life [10,11,23]. Despite these encouraging findings, there has been limited practice change among HF providers [30]. Probably owing to the fact that iron replacement therapy in HF patients remains a category II-A AHA treatment guideline recommendation and is only recommended after completing workup for other causes of ID [13]. There is also confusion about the appropriate time to administer iron therapy (inpatient vs outpatient) [15]. Practitioners that have taken up this recommendation resort to outpatient administration which can sometimes be complicated by insurance coverage and transportation. The recent Ponikowski et al. trial supports in-patient administration of IV iron in patients admitted with acute HF and their results show that this practice is both safe, cost-effective and clinically beneficial [17]. Future studies should focus on evaluating reasons for the slow adoption of IV iron therapy in HF patients with ID.

All-Cause Mortality

Our study did not show any statistically significant difference in the rates of all-cause mortality in HF patients treated with IV iron compared to placebo. This finding is consistent with prior meta-analyses on this subject [26,29]. Several factors can explain this negative finding. First, only 9.95% of the total cohort had mortality outcomes (221/2,221). It is possible that a relatively small sample size masked the potential mortality difference. Second, follow up period of included studies were relatively short and might not have been long enough to observe mortality differences between groups. With longer follow up we anticipate that there will be some future mortality benefit owing to reduced rates of HF hospitalization as studies have shown that recurrent hospitalizations in HF patients reduces length of life [31]. Applying this data, we can extrapolate that it is likely that studies with larger sample size and longer follow-up time may effectively capture mortality benefit of administering IV iron to HF patients. This is an area for further research.

Improved Hematic Values, NYHA Class and LVEF

The goal of treatment in HF remains, preventing progression, and symptomatic improvement which in turn improves prognosis and quality of life [32]. NYHA functional class is a known marker of functional capacity in HF patients [12]. Anker et al. trial was designed solely to evaluate patients’ functional capacity using NYHA class and other parameters [22]. Their results showed that IV iron therapy in HF patients with ID leads to improved functional capacity [22]. The results of this meta-analysis support this finding. Newer trials like the Ponikowski et al. trial used the six-minute walk test to evaluate functional capacity with similar results [17].

LVEF is an important treatment target since higher LVEF is associated with a better prognosis in HF patients [33]. Our results show that while IV iron therapy is associated with increased LVEF, the mechanism for this is unknown. Further trials are needed to evaluate the mechanism by which IV iron improves LVEF. As expected, treatment with IV iron led to an increase in hematic values. This is the general consensus among most studies evaluating the use of IV iron therapy in HF patients.

Strengths and limitations

Our study represents the largest number of meta-analyzed HF patients being treated with IV iron therapy. It updates recent meta-analysis and further solidifies available evidence for the use of IV iron in HF patients. We ensured reliability of our results by using a random effects model and performing multiple sensitivity analysis to evaluate the heterogeneity and consistency of the results. 

Our study has several limitations. First, our study incorporates studies with various IV iron preparations and two studies with IV iron and EPO combination regimen, although exclusion of Comín-Colet et al. and Silverberg et al. from the analysis did not impact our results [16,21]. Second, included studies have a relatively short follow up duration. Future studies incorporating longer follow up time is needed to verify the potential long-term effects of IV iron treatment. Third, among the included studies, all but one study included only patients with EF<45% [15]. Hence, our results cannot be applied to patients with HF with preserved EF. Fourth, most studies incorporated NYHA class II-III patients. Hence, we cannot generalize our results to NYHA class IV HF patients. Finally, it is unclear if these benefits are specific to a particular type of cardiomyopathy given that the included studies did not stratify patients based on the etiology of their cardiomyopathies.

## Conclusions

The results of this meta-analysis of nine studies revealed that treatment with IV iron compared to placebo was associated with a significant increase in serum ferritin, transferrin saturation and Hb levels. Furthermore, our results revealed that IV iron therapy in HF patients was associated with a reduction in rates of HF hospitalization and NYHA class.

This study represents the largest number of meta-analyzed patients evaluating clinical outcomes, hematic values, symptomaticity and cardiovascular parameters adding to the growing body of evidence advocating for the use of IV iron therapy in HF. 

Future studies exploring the use of IV iron in HF patients with longer follow-up times are needed to further explore the clinical and mortality benefits of IV iron in HF patients with ID.
